# Multi-Person Tracking and Crowd Behavior Detection via Particles Gradient Motion Descriptor and Improved Entropy Classifier

**DOI:** 10.3390/e23050628

**Published:** 2021-05-18

**Authors:** Faisal Abdullah, Yazeed Yasin Ghadi, Munkhjargal Gochoo, Ahmad Jalal, Kibum Kim

**Affiliations:** 1 Department of Computer Science, Air University, Islamabad 44000, Pakistan; 191633@students.au.edu.pk (F.A.); ahmadjalal@mail.au.edu.pk (A.J.); 2 Department of Computer Science and Software Engineering, Al Ain University, Abu Dhabi 122612, United Arab Emirates; Yazeed.ghadi@aau.ac.ae; 3 Department of Computer Science and Software Engineering, United Arab Emirates University, Al Ain 15551, United Arab Emirates; mgochoo@uaeu.ac.ae; 4 Department of Human-Computer Interaction, Hanyang University, Ansan 15588, Korea

**Keywords:** bat optimization, human crowd behavior (HCB), improved entropy (IE), Jaccard similarity, multi-person counting, particles gradient motion (PGM), speeded up robust features (SURF)

## Abstract

To prevent disasters and to control and supervise crowds, automated video surveillance has become indispensable. In today’s complex and crowded environments, manual surveillance and monitoring systems are inefficient, labor intensive, and unwieldy. Automated video surveillance systems offer promising solutions, but challenges remain. One of the major challenges is the extraction of true foregrounds of pixels representing humans only. Furthermore, to accurately understand and interpret crowd behavior, human crowd behavior (HCB) systems require robust feature extraction methods, along with powerful and reliable decision-making classifiers. In this paper, we describe our approach to these issues by presenting a novel Particles Force Model for multi-person tracking, a vigorous fusion of global and local descriptors, along with a robust improved entropy classifier for detecting and interpreting crowd behavior. In the proposed model, necessary preprocessing steps are followed by the application of a first distance algorithm for the removal of background clutter; true-foreground elements are then extracted via a Particles Force Model. The detected human forms are then counted by labeling and performing cluster estimation, using a K-nearest neighbors search algorithm. After that, the location of all the human silhouettes is fixed and, using the Jaccard similarity index and normalized cross-correlation as a cost function, multi-person tracking is performed. For HCB detection, we introduced human crowd contour extraction as a global feature and a particles gradient motion (PGD) descriptor, along with geometrical and speeded up robust features (SURF) for local features. After features were extracted, we applied bat optimization for optimal features, which also works as a pre-classifier. Finally, we introduced a robust improved entropy classifier for decision making and automated crowd behavior detection in smart surveillance systems. We evaluated the performance of our proposed system on a publicly available benchmark PETS2009 and UMN dataset. Experimental results show that our system performed better compared to existing well-known state-of-the-art methods by achieving higher accuracy rates. The proposed system can be deployed to great benefit in numerous public places, such as airports, shopping malls, city centers, and train stations to control, supervise, and protect crowds.

## 1. Introduction

Multi-person tracking is currently one of the most essential and challenging research topics in the computer vision community [1,2,3,4,5,6,7,8,9]. Because of the common availability of high-quality low-cost video cameras and considering the inefficiency of manual surveillance and monitoring systems, automated video surveillance is now essential for today’s crowded and complex environments. To monitor, control, and protect crowds, accurate information about numbers plays a vital role in operational and security efficiencies [10,11,12,13,14,15,16]. The counting and tracking of many persons is a challenging problem [17,18,19,20,21,22,23,24,25] due to occlusions, the constant displacement of people, different perspectives and behaviors, varying illumination levels, and because, as the crowd gets bigger, the allocation of pixels per person decreases.

A primary concern in surveillance and monitoring systems is to identify human crowd behaviors and supervise the crowd to prevent disasters and unforeseen events [26,27,28,29,30,31,32,33,34]. The analysis of human behavior in crowded scenes is one of the most important and challenging areas in current research [35,36,37,38,39,40,41,42,43]. Traditional visual surveillance systems that depend purely on manpower to analyze videos is inefficient because of the enormous number of cameras and screens that require monitoring, human fatigue due to time spent on lengthy monitoring periods, paucity of essential fore-knowledge and training in what to look for, and also because of the colossal amount of video data that is generated per day. Such issues necessitate an automated visual surveillance system that can reliably detect, isolate, analyze, identify, and alert responders to unusual events in real time. Automated surveillance systems seek to detect human behaviors automatically in crowded scenes, and it has many potential applications, such as security, care of the elderly and infirm, traffic monitoring, inspection tasks, military applications, robotic vision, sports analysis, video surveillance, and pedestrian traffic monitoring [44,45,46,47,48,49,50,51,52].

In this research article, we propose a robust new particles-based approach for multi- person counting and tracking, which addresses the problematic fact that, as the density of a crowd increases, the number of pixels allocated per human decreases. By using our particles-based approach, we were able to count and track multiple persons in crowded scenes and efficiently deal with occlusions, arbitrary movements, and overlaps. We also propose a new approach for crowd behavior detection using an improved entropy classifier based on the fusion of global and local descriptors extraction. First of all, we applied pre-processing steps on extracted video frames for noise removal, edge detection, and contrast adjustment, then human/non-human detection was performed using multi-level thresholding and morphological operations. We applied a distance algorithm for human silhouette extraction. After that, our work involved two facets: (i) multi-people tracking and (ii) crowd behavior detection. In the multi-person tracking phase, we first verified the extracted silhouettes by a particles force model, then we converted extracted foreground objects into particles, and, using physics phenomena of the mutually interacting particles force model, non-human objects were discarded. As every extracted human silhouette is a collection of particles, by treating groups of particles that make one silhouette as a cluster, we performed labeling and cluster estimation using a K-nearest neighbors search algorithm to count the persons. We then fixed the human silhouettes with a unique integer ID, and, using normalized cross correlation as a cost function and the Jaccard similarity index, multi-person tracking was performed. However, for crowd behavior detection, we used a fusion of global and local descriptors, that is, after foreground extraction, we extracted a human crowd contour as a global descriptor and a particles gradient motion (PGM) descriptor, along with geometric and speeded up robust features (SURF) as local descriptors. Using this fusion of global and local descriptors, bat optimization was then applied for optimal descriptors. Finally, by using Shannon’s information entropy theory [53], we introduced an improved entropy classifier to detect crowd behavior.

Experimental results show that our proposed system performed better compared to existing well-known state-of-the-art methods. The proposed system has huge potential applications, such as crowd density estimation, security, care of the elderly and vulnerable, sports analysis, inspection tasks, military applications, robotic vision, video surveillance, and pedestrian traffic monitoring. The major contributions of this paper can be highlighted as follows:

1. We propose a new particles force model for human silhouettes verification, which is a necessary step for accurate counting and tracking of multiple persons in crowded scenes.
2. We developed a novel particles gradient motion local descriptor and human crowd contour as a global descriptor, while the fusion of global and local features was used for crowd behavior detection.
3. We designed an improved entropy classifier to analyze contextual information and classify crowd behavior in a more efficient manner.
4. We evaluated the performance of our proposed multi-person tracking approach on a publicly available benchmark PETS2009 dataset while crowd behavior detection performance was evaluated on the publicly available benchmark UMN dataset and the proposed method was fully validated for efficacy, surpassing other state-of-the-art methods, including deep learning.

The remaining structure of this paper was arranged as follows: Section 2 describes related work. A detailed overview of the proposed model for multi-person tracking and crowd behavior detection is mentioned in Section 3, which includes preprocessing, human silhouettes extraction, the particles force model, multi-person counting, multi-person tracking, global and local features extraction, bat optimization, and an improved entropy classifier. In Section 4, we evaluate the performance of our proposed approach on a publicly available benchmark dataset and give a detailed comparison of our proposed approach with other state-of-the-art methods. Lastly, in Section 5, we sum up the paper and outline future directions.

## 2. Related Work

During the last few years, several algorithms and systems have been developed by different researchers for crowd counting, tracking, and human behavior detection [54,55,56,57,58,59,60,61,62]. Here, we divide the related work into two parts, namely, human crowd behavior detection systems and multi-person counting and tracking systems.

### 2.1. Crowd Behavior Detection Systems

Many contributions have been proposed to describe crowd behavior using various models [63,64,65,66,67,68,69]. Crowd behavior detection is a challenging problem due to the arbitrary movements of individuals and groups, partial or full occlusions, different outlooks and behaviors, posture changes, and composite backgrounds [70,71,72,73,74,75,76]. To detect human behaviors automatically in crowded areas, S. Wu et al. in [77] constructed a density function of optical flow based on class-conditional probability and described the motion of crowds using divergent centers and potential destinations so that anomalies can be detected on the basis of a Bayesian framework. However, the system is not effective for arbitrary movements or overlaps. S. Choudhary et al. in [78] proposed a SIFT feature extraction technique, along with a Genetic Algorithm for optimal feature extraction; anomalies were detected by checking feature set movement behaviors. Their proposed system has a very high computational processing demand. Direkoglu et al. in [79] used a one-class SVM, along with features based on optical flow to detect crowd behavior; their system is limited by the accuracy limitations of optical flow estimation. W. G. Aguilar et al. in [80] introduced a moved-pixels density-based statistical modeling approach for detecting abnormal crowd behavior. This system has low computational cost, but the efficiency decreases with increasing complexity of the situation being monitored, e.g., serious occlusions. A. Shehzed et al. in [81] first detected humans and then the gaussian smoothing technique was used to detect anomalous behavior; however, the accuracy of the system decreases with illumination changes and occlusions because thresholding is used for detection. W. Ren et al. in [82] introduced a behavior entropy model for detecting abnormal crowd behavior using spatio-temporal information, along with behavior certainty of pixels, but the system is vulnerable to certain misclassifications due to interclass similarities. G. Wang et al. in [83] addressed the crowd behavior detection problem by using the pyramid Lucas-Kanade optical flow [84] method based on location estimation of adjacent flow; however, the proposed method is not effective for an unstructured crowd. R Mehran et al. in [85] placed a grid of particles on the image and introduced a social force model for detecting crowd behavior. Bellomo, N. et al. in [86] pursued two specific objectives: the derivation of a general mathematical structure based on appropriate developments of the kinetic theory suitable for capturing the main features of crowd dynamics and the derivation of macroscopic equations from the underlying mesoscopic description. Colombo, R.M. et al. in [87] dealt with macroscopic modelling of crowd movements, particularly how non-local interactions are influenced by walls, obstacles, and exits. An ad hoc numerical algorithm, along with heuristic evaluation of its convergence, was also provided. Khan, S.D. et al. in [88] proposed scale estimation network SENet and head detection network. The SENet takes the input image and predicts the distribution of scales (in terms of histogram) of all heads in the input image, which are later on classified by a detection network.

### 2.2. Multi-Person Counting and Tracking Systems

True foreground extraction, i.e., human pixels, is only one of the primary steps for accurate counting and tracking of humans in crowded scenes [89,90,91,92,93]. Several approaches and systems have been introduced by many researchers for multi-person counting and tracking. In [94], S. Choudri et al. proposed a pixels-based people counting model using the fusion of a pixel map-based algorithm along with human detection to count only human classified pixels. They applied a depth map, image segmentation, and a human presence map that was updated with a human mask for the purpose of counting people; however, the system has misclassification problems due to interclass similarities. H. Chen et al. in [95] proposed a new color and intensity patch segmentation approach for tracking and detection of human body parts and for the full body. They applied fusion of color space segmentations for the detection of body parts and for the full body. For tracking, based on the velocity of a target, they adaptively selected the track gate size. A target’s likely forward position was predicted based on the target’s previous velocity and direction. The proposed algorithm achieved satisfactory results only when the count of peoples was limited in the view, i.e., efficiency decreases as the crowd increases. In [96], J. Garcia et al. introduced a head tracking-based directional people counter. Using several circular patterns and preprocessing steps, people’s heads were detected. For the tracking application, a Kalman filter was used, and counting was achieved on the bases of head detection and tracking. The effectiveness of the proposed algorithm decreases during serious occlusions, arbitrary movements, and overlaps. M. Vinod et al. in [97] introduced object tracking and counting using new morphological techniques. The frame-difference technique, followed by morphological processing and region growing, was used for counting people. Moving objects were extracted by determining their movements, and then tracking was performed using color features. As the illumination of the scene changed, the efficiency of the proposed algorithm decreased. G. Liu et al. in [98] proposed a tracker based on a correlation filter. Kalman filter applications were used for tracking. They designed a tracker that detects numerous positions and alternate templates. However, the system was not efficient in dealing with complex situations, such as occlusions and random movements. E. Ristani et al. in [99] used deep learning to track multi-persons. Using CNN, they extracted features and then introduced a weighted triple loss strategy to assign weights during training. Their system was computationally complex, and a huge dataset was essential for training. H. Xu et al. in [100] located humans by their shoulders and heads, and, for tracking, they used trajectory analysis and the Kalman filter, but the system was not effective for arbitrary movements or overlaps.

## 3. Proposed System Methodology

**Figure 1:**
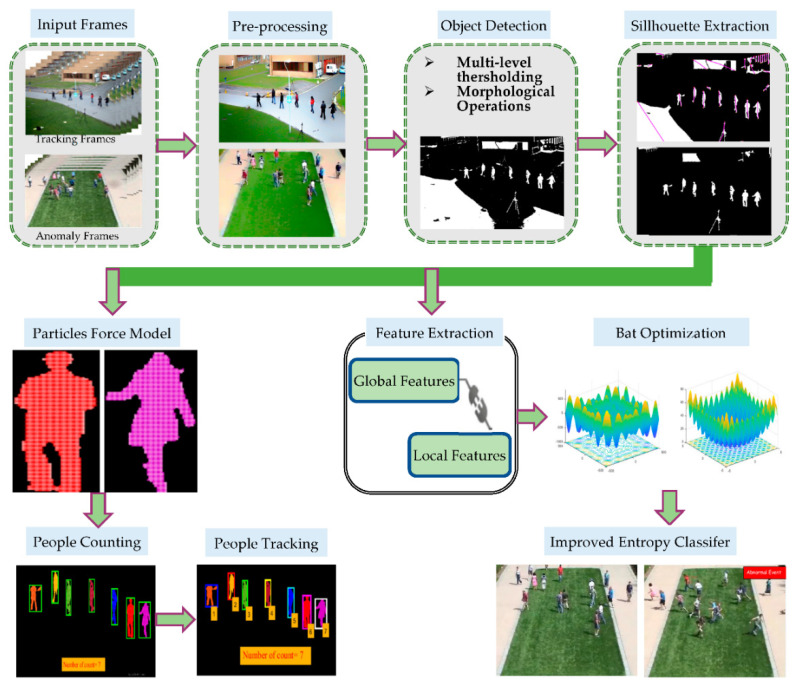
Synoptic schematics of the proposed Multi-Person Tracking and Crowd Behavior Detection system.

This section elaborates our proposed methodology for multi-person tracking and crowd behavior detection. We propose a robust multi-person tracking system based on a particles force model and human crowd behavior detection system using an improved entropy classifier with spatio-temporal and particles gradient motion descriptors. In the proposed system, the first step is the preprocessing of extracted video frames from a static camera. Secondly, object detection is transacted using multi-level thresholding, morphological operations, and labeling. Thirdly, for human silhouette extraction, a distance algorithm is applied, and non-human filtering is performed on all extracted labeled objects. At this stage, we administered our work into two streams: the first was for multi-person counting and tracking, where we first performed a human silhouette verification step by converting extracted objects into particles and a robust particles force model was introduced for human silhouette verification. In the next step, after verification of human silhouettes, as all verified human silhouettes are a collection of particles, by treating each group of particles as a cluster we performed labeling and cluster estimation using a K-nearest neighbors searching algorithm for multi-person counting. After that, for multi-person tracking, the position of each detected human silhouette was then located and locked by assigning an integer ID for temporally fixing each human silhouette in the full video, and detected fixed humans were tracked using a Jaccard Similarity Index. However, in the second facet, for crowd behavior detection, the extracted foreground objects were passed through a feature extraction step and multiple distinguishable global and local features were extracted from every frame. After that, all the extracted features were standardized using the bat optimization algorithm. Lastly, in the classification phase, an improved entropy classifier was proposed for detection of crowd behavior. Figure 1 depicts the synoptic schematics of our proposed system.

### 3.1. Pre-Processing

**Figure 2:**
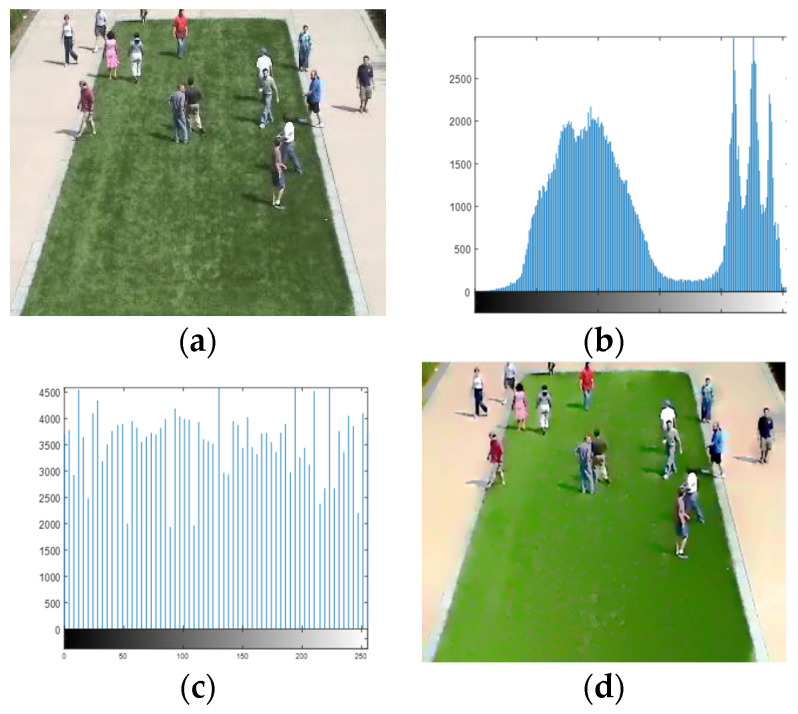
Preprocessing steps. (**a**) Original color frame of a video, (**b**) histogram of original image, (**c**) histogram of enhanced image, and (**d**) enhanced image.

During image pre-processing, color frames were extracted from a static video camera E=[*f*_1_,*f*_2_,*f*_3,_ …, *f_Z_*], where Z is the total number of frames. These color images were then passed through a Laplacian filter to reduce the noise and sharpen the edges. A Laplacian filter was applied using Equation (1):

(1) $$\nabla^{2}f=\frac{\partial^{2}f}{\partial^{2}x}+\frac{\partial^{2}f}{\partial^{2}y}$$

where $\nabla^{2}f$ is the 2nd order derivative for obtaining the filtered mask. However, a pure Laplacian filter did not produce an enhanced image, thus, to achieve the sharpened enhanced image, we subtracted the Laplacian outcome from the original image using Equation (2):

(2) $$g\left(x,y\right)=f\left(x,y\right)-\left[\nabla^{2}f\right]$$

where the g(x,y) is the sharpened image and f(x,y) is the input image. After obtaining the sharpened image g(x,y), histogram equalization was performed on the sharpened image in order to adjust the contrast of an image using Equation (3):

(3) $$s_{k}=T\left(r_{k}\right)=\left(L-1\right)\sum_{j=0}^{k}p_{r}\left(r_{j}\right)\;\;\;k=0,\;1,\;2,\;\ldots ,\;L-1$$

where variable *r* denotes the intensities of an input image to be processed. As usual, we assumed that *r* is in the range [0 *L* − 1], with *r* = 0 representing black and *r* = *L* − 1 representing white, while *s* represents the output intensity level after intensity mapping for every pixel in the input image, having intensity *r.* However, $p_{r}\left(r\right)$ is the probability density function (PDF) of *r*, where the subscript on *p* were used to indicate that it was a PDF of *r*. Thus, a processed (output) image was achieved using Equation (3) by mapping each pixel in the input image with intensity $r_{k}$ into a corresponding pixel with level $s_{k}$ in the output image, as shown in Figure 2.

### 3.2. Human Silhouettes Extraction

**Figure 3:**
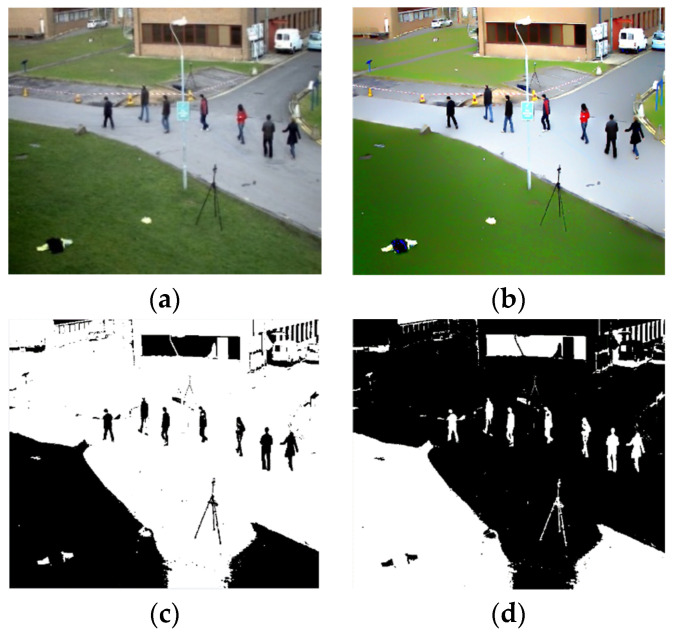
Object detection steps. (**a**) Original color frame of a video, (**b**) enhanced image, (**c**) binary image after multi-level thresholding, and (**d**) inverse of a threshold image.

After obtaining the preprocessed frames, we performed human/non-human detection by performing multi-level thresholding using Equation (4), as depicted in Figure 3c.

(4) $$th\left(x,y\right)=\{\begin{array}{cc} 1 & \;\mathrm{if}\;\;\;\;l\left(x,y\right)>t_{1},t_{2},t_{3}\\ 0 & \;\mathrm{otherwise}\; \end{array}$$

where th(x,y) is the threshold image and $t_{1},t_{2},t_{3}$ are the applied thresholds that are defined by Otsu’s procedure. In order to extract more useful information, the resultant binary image was inverted using a point processing operation that subtracts every pixel of an image from the maximum level of the image, as shown in Equation (5).

(5) C(x,y)=1−th(x,y)

where C(x,y) is the inverted image, as shown in Figure 3d, and th(x,y) is the binary image with a maximum level of 1. After obtaining the human/non-human binary foreground frames, we performed morphological operations to remove imperfections in the inverted image C. For the removal of small unwanted objects, erosion was performed, and then, to fill small holes while preserving the size and shape of objects, morphological closing was performed. Every object in image *C* was first eroded using erosion as represented in Equation (6) and then dilated using Equation (7), after which the dilated image was eroded again using the disk-shaped structuring element, as shown in Equation (8).

(6) $$m\left(x,y\right)=\{\begin{array}{cc} 1 & \;\mathrm{if}\;\;\;\;\;\;\;\;\;\;S\;\mathrm{fits}\;C\\ 0 & \;\mathrm{otherwise}\; \end{array}$$

(7) $$m\left(x,y\right)=\{\begin{array}{cc} 1 & \;\mathrm{if}\;\;\;\;\;\;\;\;\;\;S\;\mathrm{hits}\;C\\ 0 & \;\mathrm{otherwise}\; \end{array}$$

(8) Mo=(C ⊖ S)((C⊕S)⊖S)

where C represents the input inverted image and S is the disk-shaped structuring element used for erosion and dilation, while Mo is the resultant image. The erosion of C by S is denoted as (C ⊖ S); however, the dilation of C by S is denoted as (C⊕S). After morphological operations, all the objects in the image were grouped and labeled, which helped in extracting and uniquely analyzing every object that was required for human silhouette extraction.

**Figure 4:**
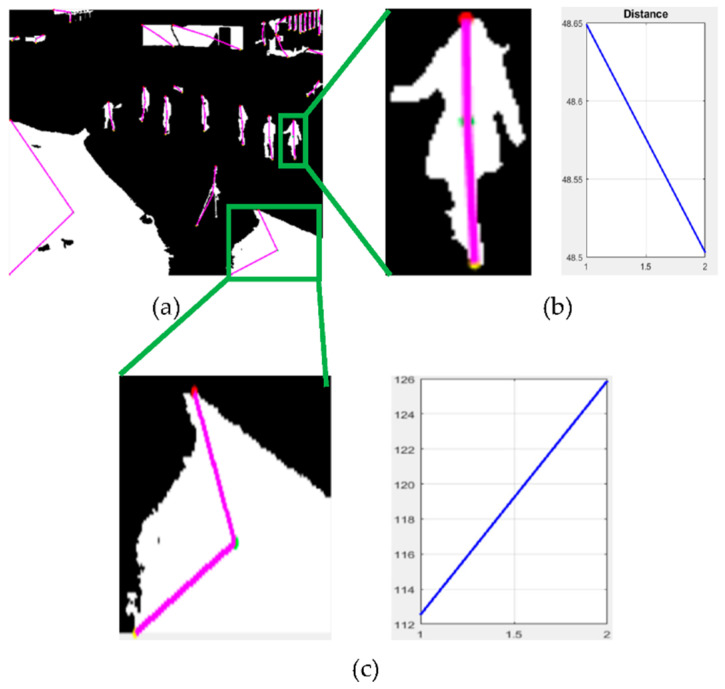
Human silhouette extraction. (**a**) Distance algorithm from the center to two extreme points for every object, (**b**) single silhouette extracted uniquely through labeling, along with its distance graph, and (**c**) a single non-silhouette, along with its distance graph.

After human/non-human detection, for human silhouette extraction, we calculated the center and extreme points of each of the labeled objects of *M_o_*, then we extracted each object one by one, and the distance from center to two extreme points was calculated for every object for non-human filtering, as shown in Figure 4. The same procedure was adopted for the frames from frame 1 to frame Z.

After calculating the distances, those objects whose distances were greater than the set threshold were discarded using Equation (9), and only silhouettes resembling humans were retained.

(9) $$E_{h}=\{\begin{array}{cc} 0 & \;\mathrm{if}\;d_{1}>T\cap d_{2}\;>\;T\\ 1 & \;\mathrm{otherwise}\; \end{array}$$

where the distance from the center to one extreme point is denoted by $d_{1}$, the center to the other extreme point distance is represented by $d_{2}$, *T* is the set threshold and *E_h_* is the resultant image. After human silhouette extraction, most of the non-human objects were discarded by the distance algorithm; however, some non-human objects that resembled human objects remained.

### 3.3. Multi-Person Tracking

For accurate human tracking, the extraction of the true foreground, i.e., human pixels only, is a primary step. Thus, after application of the distance algorithm (mentioned in Section 3.2) for multi-person tracking, we performed the human silhouette verification step using the particles force model, and then the multi-person counting and tracking steps were executed.

#### 3.3.1. Human Silhouettes Verification: Particles Force Model

**Figure 5:**
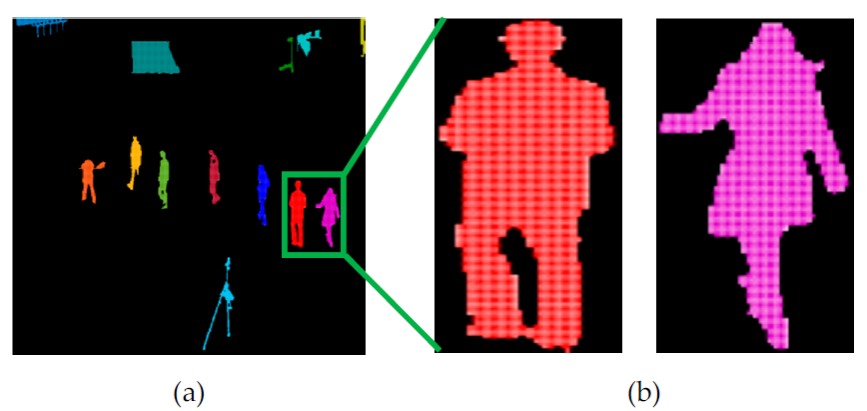
The particles force model. (**a**) Particle conversion of every extracted silhouette and (**b**) magnified view of particle conversion.

We present a robust particles force model for human silhouette verification. First of all, every extracted labeled silhouette was converted into particles, as shown in Figure 5a. We treated all pixels as fluid particles, thus, every extracted silhouette was a collection of many particles, as depicted in the magnified view in Figure 5b. Therefore, in our designed method, each silhouette was represented by a set of particles *Q* = [$p_{1}$,$\;p_{2}$,$\;p_{3}$, *…*,$\;p_{N}$], where *N* is the total number of particles in one silhouette.

**Figure 6:**
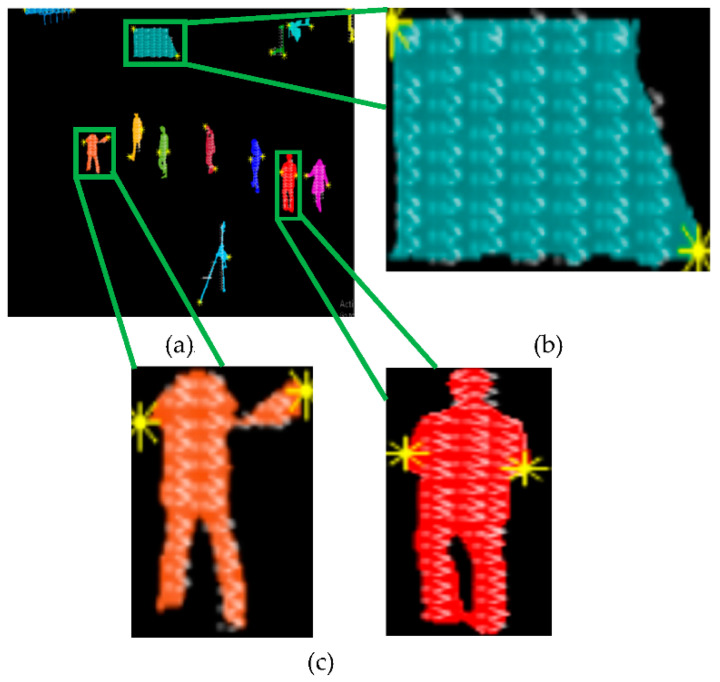
Particles force model. (**a**) Interacting force between two particles (**b**) for non-human silhouettes and (**c**) human silhouettes.

**Figure 7:**
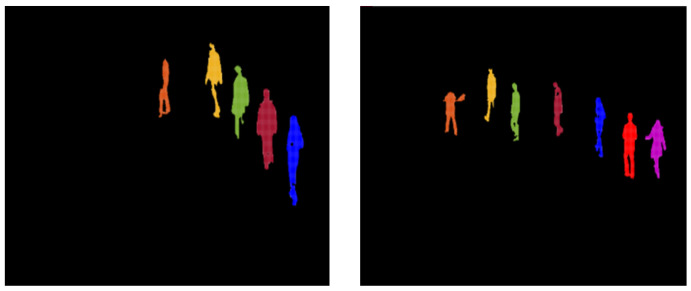
A few examples of verified multi-human silhouettes.

We know from physics that, in solids, particles do not have enough kinetic energy to overcome the strong forces of attraction, called bonds, which attract the particles toward each other. Using this physics phenomenon, we found the force of attraction between particles of every extracted silhouette, as shown in Figure 6:

For simplicity, we found the force of attraction between only two mutually interacting particles using Equation (10) in all frames from 1 to Z.

(10) $$F_{i}=\frac{p_{1}p_{2}}{r^{2}}$$

where *i* is in the range [1 *E*] with *E*, representing the maximum number of silhouettes per frame, while $F_{i}$ is the force of attraction between particle $p_{1}$ and $p_{2}$ of the *i*th silhouette and *r^2^* is the square of Euclidian distance between particles $p_{1}$ and $p_{2}$. After calculating the force between particles of every silhouette in all video frames, we discarded those silhouettes whose force of attraction was static in frame t and frame *t* + 1 using Equation (11):

(11) $$H_{s}=\{\begin{array}{cc} 1 & \;\mathrm{if}\;\;\frac{dF_{i}}{dt}>0\\ 0 & \;\mathrm{otherwise}\; \end{array}$$

where $\frac{dF_{i}}{dt}$ represents the change in attraction force between particles of every *i*th silhouette, with respect to time between frames *t* to *t +* 1. After application of the particles force model, we only retained human silhouettes in each frame, as depicted in Figure 7:

#### 3.3.2. Multi-Person Counting

**Figure 8:**
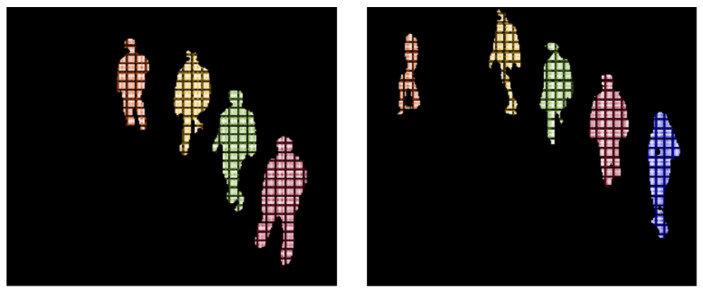
Human contours for cluster estimations.

**Figure 9:**
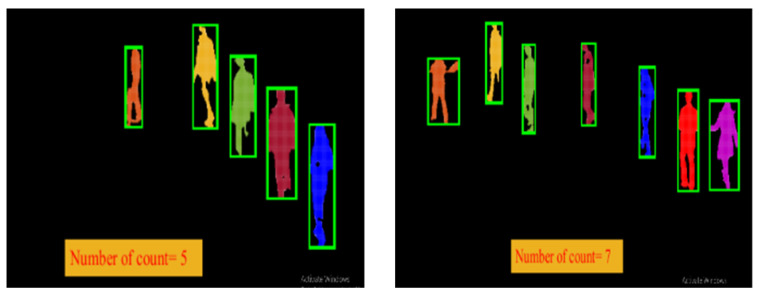
Sample frames of multi-person counts at different time intervals.

After extraction of the verified human silhouettes, to count these detected humans silhouettes, which consist of a set of particles, we performed cluster estimation. Since every silhouette is a collection of particles, the group of particles that makes one silhouette was treated as one cluster, and, by using the K-nearest neighbor search algorithm, cluster estimation was performed on every frame, as depicted in Figure 8:

After that, we labeled clusters in all frames, as shown in Equation (12), and, to make them appear visually, we drew green bounding boxes around each cluster. Thus, by performing cluster estimation and labeling, we counted all the extracted human silhouettes, as shown in Figure 9:

(12) $$I_{c}=L_{m}p_{N}$$

where $p_{N}$ is the total number of particles in one cluster (the total number of particles in each cluster varies from cluster to cluster and the number of clusters in each frame varies from frame to frame), while $L_{m}$ represents the label of cluster *m* and $I_{c}$ is the resultant extracted labeled cluster that was treated as one silhouette and was considered in counting.

#### 3.3.3. Multi-Person Tracking

The goal of person tracking is to establish correspondence between individuals across frames. Thus, to establish correspondence between persons in frame *t* and frame *t* + 1, we calculated the position and velocity of every detected human silhouette in all frames. In our model, we assumed that people can enter or leave the scene, thus, for temporally fixing of all humans across frames, the position of each human silhouette was located and locked by assigning a unique integer ID, which was fixed to that particular silhouette in all frames. The states of all the predicted persons in frame *F_t_* were stored in a structure and matched with the states of frame *F_t_*_+ 1_, while the detected fixed human silhouettes were tracked using the Jaccard similarity index.

(13) $$S_{t}=\sum_{i=1}^{n}I_{Li}$$

**Figure 10:**
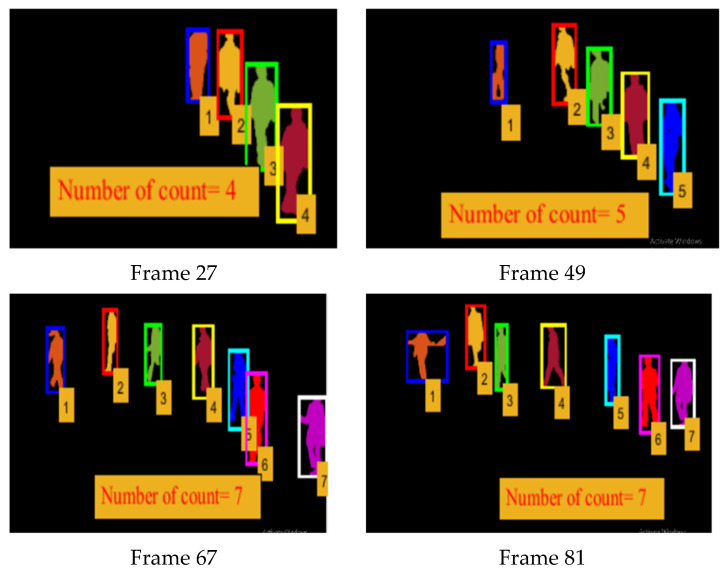
Sample frames of multiple human silhouette-fixing and tracking at different time intervals.

**Figure 11:**
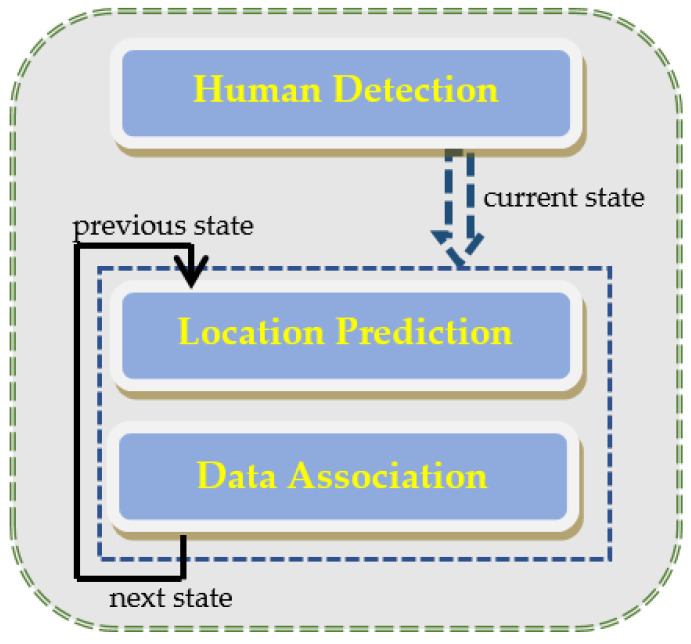
Key steps involved in multi-person tracking.

While using data association and cross-correlation as a cost function, detected and predicted persons were associated in consecutive frames, as represented in Figure 10. The root steps involved in multi-person tracking are illustrated in Figure 11.

### 3.4. Crowd Behavior Detection

Understanding that accurate crowd behavior requires robust global and local feature extraction [101,102,103], along with a potent decision-making classifier, for crowd behavior detection after applying the distance algorithm (mentioned in Section 3.3), the extracted silhouettes were passed through the feature extraction step and multiple distinguishable global and local features were extracted for every frame. Next, bat optimization was applied for optimal feature extraction and decisions were made by the improved entropy classifier. 

#### 3.4.1. Global-Local Descriptors

For the global-local descriptor, we used a fusion of global and local image properties. In global features, we described the visual content of the whole image and we had the ability to represent an image with a single vector. Here, we extracted the crowd contour as a global feature. For local features, we used our newly proposed particles gradient motion features, geometric features, and speeded up robust feature (SURF) [104]. For local features, we extracted interest points and represented them as a set of vectors that respond more vigorously to clutter and occlusions.

**Figure 12:**
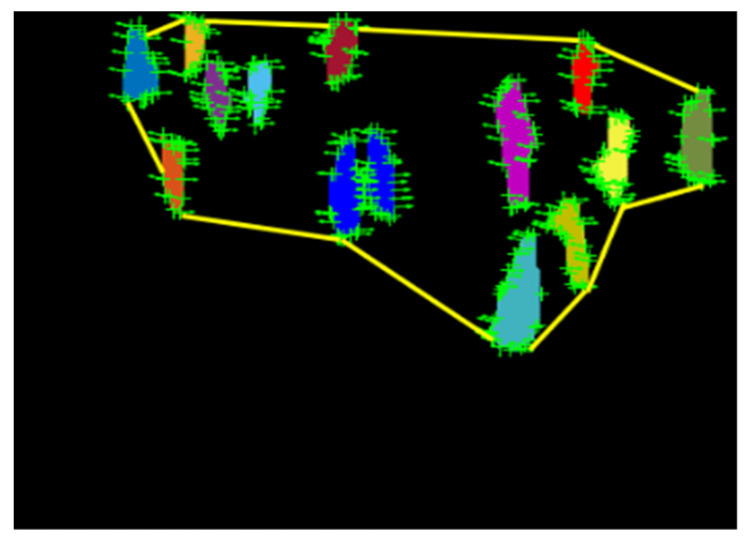
Extraction of human crowd contour as a global feature.

Initially, in global features, we found the center of each human and considered all the humans in the scene as a vertex; this can be denoted as *P* = {*P*1, *P*2, *…*, *Pn|Pi* = (*Xi*, *Yi*)}, where *P* represents the whole human crowd in the scene, considered as a set of vertices, and (*Xi*, *Yi*) are the coordinates of the *i*th human. We considered only those humans that were at the extreme points and joined them with a line, forming the biggest graph, covering all extreme vertices, as shown in Figure 12. The graph represented the human crowd contour, and thus, the variations in the shape of a graph threw a flash on variations in the outer area of the human crowd, i.e., on global changes. To measure the variations in the crowd contour, we compared the contour temporally by integrating over all of the pixels of the contour. In general, we defined the (*p*, *q*) moment of a contour as in Equation (14):

(14) $$m_{p,q}=\sum_{x}^{n}\sum_{y}^{n}I\left(x,y\right)x^{p}y^{q}$$

where I(x,y) is the intensity of the pixels in coordinate (x,y). Here, p is the *x*-order and q is the *y*-order, whereby, order means the power to which the corresponding component is taken in the sum just displayed. The summation is over all of the pixels of the contour boundary (denoted by *n* in the equation). It then follows immediately that, if p and q are both equal to 0, then the $m_{0,0}$ moment is actually just the length in pixels of the contour. The moment computation just described gives some rudimentary characteristics of a contour that can be used to compare two contours.

**Figure 13:**
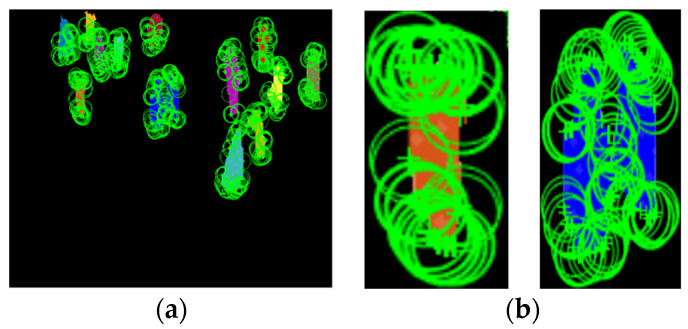
(**a**) SURF features for all human silhouettes and (**b**) magnified view of SURF features for two human silhouettes.

**Figure 14:**
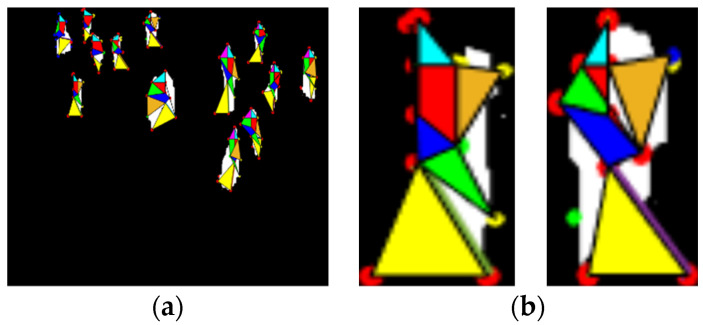
(**a**) Geometric features for all human silhouettes. (**b**) Magnified view of geometric features for two human silhouettes.

In the SURF descriptor [105], we computed distinctive invariant local features, which detected the interest points and elaborate features that depict some invariance to image noise, rotation, direction, scaling, and changes in illumination. Using SURF, we computed 75 local points for every human silhouette in an image, and thus, for every frame, we had 1050 SURF descriptors in a set of vectors, as shown in Figure 13:

In geometric local features, we first identified the skeleton joints of every human silhouette in each frame using a skeleton model, and then, by considering skeleton joints as vertices, we drew poly-shapes and triangles with three or four vertices. By using the left arm, neck, left shoulder, and torso, a left polygon wing was drawn and filled with a color. Similarly, a right polygon wing was drawn and filled with different colors using the right arm, neck, torso, and right shoulder. Additionally, the torso area, lower area, left shoulder triangles, and right shoulder triangles were drawn, as depicted in Figure 14. The areas enclosed under these polygons were analyzed frame by frame, and on the basis of angle differences and area size, normal and abnormal behaviors of human crowds were detected. Algorithm 1 depicts the overall procedure used for the extraction of the strongest body points for human silhouettes.

**Figure 15:**
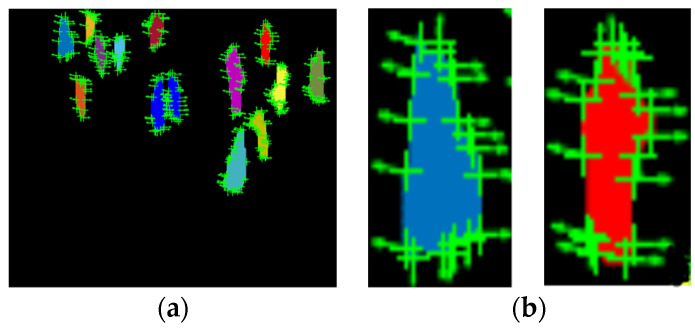
(**a**) Particles gradient motion descriptors for all human silhouettes and (**b**) magnified view of PGM for two human silhouettes.

In particles gradient motion (PGM), we first converted every human silhouette into particles and then only those particles that were on the human contour were considered, and their interaction force was calculated. Generally, every pedestrian in a crowd has a desired direction and velocity *v_i_^d^*, calculated using Equation (16). However, in crowded scenes, because of the presence of multiple persons, individual movements are limited, and the actual velocity of each pedestrian *v_i_* is different from their respective expected motion. The actual velocity of particles is calculated using Equation (15).

(15) $$v_{i}=F_{avg}\left(x_{i},\;y_{i}\right)$$

where $F_{avg}\left(x_{i},\;y_{i}\right)$ is the *i*th particle average optical flow in the coordinate $\left(x_{i},\;y_{i}\right)$. We calculated the desired velocity *v_i_^d^* of particles as:

(16) $${v_{i}}^{d}=\left(1-w_{i}\right)\;F\left(x_{i},\;y_{i}\right)+\;w_{i}F_{avg}\left(x_{i},\;y_{i}\right)$$

where $F\left(x_{i},\;y_{i}\right)$ represents *i*th particle optical flow with coordinates$\;\left(x_{i},\;y_{i}\right)$ and $w_{i}$ is the panic weight parameter. The pedestrian *i* displays vanity behaviors as $w_{i}$ *→* 0 and collective behaviors as $w_{i}$ *→* 1. Linear interpolation was used for the enumeration of efficient optical flow and the adequate average flow field of particles. Thus, on the basis of the actual velocity and the desired velocity, we can calculate the interaction force using Equation (17):

(17) $$F_{\mathrm{int}}=\frac{1}{T}\;({v_{i}}^{d}\;-\;v_{i})–\;\frac{dv_{i}}{dt}$$

where $F_{\mathrm{int}}$ is the resultant interaction force, as represented in Figure 15 and T is the relaxation parameter. When the interaction force of particles was greater than the set threshold, it was detected as an abnormal event; otherwise, it was considered to be normal.
**Algorithm 1** Extract strongest body points for human silhouettes**Input: I:** Extracted Human Silhouettes**Output:** Strongest body points, i.e., head, shoulders, legs, arms, hips/* for each connected component, extract body points.B = bwboundaries(binary_image);lbl = bwlabel(binary_image);CC2 = bwconncomp(lbl); L52 = labelmatrix(CC2);for objectidx2 = 1:CC2.NumObjectsindividualsilheouts2 = bsxfun(@times, closezn, L52 == objectidx2);[labeledImage2,numberofBlobs2] = bwlabel(individualsilheouts2,4);endAa = individualsilheouts2;/* Defining a upper, middlle and lower portion for each individual silheouts */th = thershold;rps = regionprops(Aa,’Boundingbox’, ‘Area’);**for** k = 1 to length(rps) do  w = rps(k). Boundingbox     if height > th and width > th thenupper_region = struct(‘x’,w(1), ‘y’, w(2), ‘width’,w(3), ‘height’, w(4)/5); /* head */middle_region = struct(‘x’,w(1), ‘y’, w(2) + w(4)/4, ‘width’,w(3), ‘height’, w(4)/4); /* arms */lower_region = struct(‘x’,w(1), ‘y’, w(2) + w(4)/2, ‘width’,w(3), ‘height’, w(4)/2); /* legs */j = j+1;s(j) = w;    **end**
**end**top = [x,max_y]:left = [min_x,y]:bottom = [x,min_y]:right = [max_x,y];% label the head region% Head =top pixels of upper region Right Shoulder = Bottom right pixels of upper regionLeft Shoulder = Bottom left pixels of upper regionRight arm = Right Pixels of middle region Left arm = Left Pixels of middle regionRight foot = Bottom right pixels of lower regionLeft foot = Bottom left pixels of lower region**return** Head, Shoulders, arms, foots

#### 3.4.2. Event Optimization: Bat Optimization

Optimization is a process by which the optimal solutions of a problem that satisfies and objective function are accessed [106,107,108,109]. Yang, in [110], introduced an optimization algorithm inspired by a property of bats, known as echolocation. Echolocation is a type of sonar that enables bats to fly and hunt in the dark. The bat optimization (BO) algorithm is composed of multiple variables of a given problem. Using the echolocation capability, bats can detect obstacles in the way and the distance, orientation, type, size, and even the speed of their prey.

**Figure 16:**
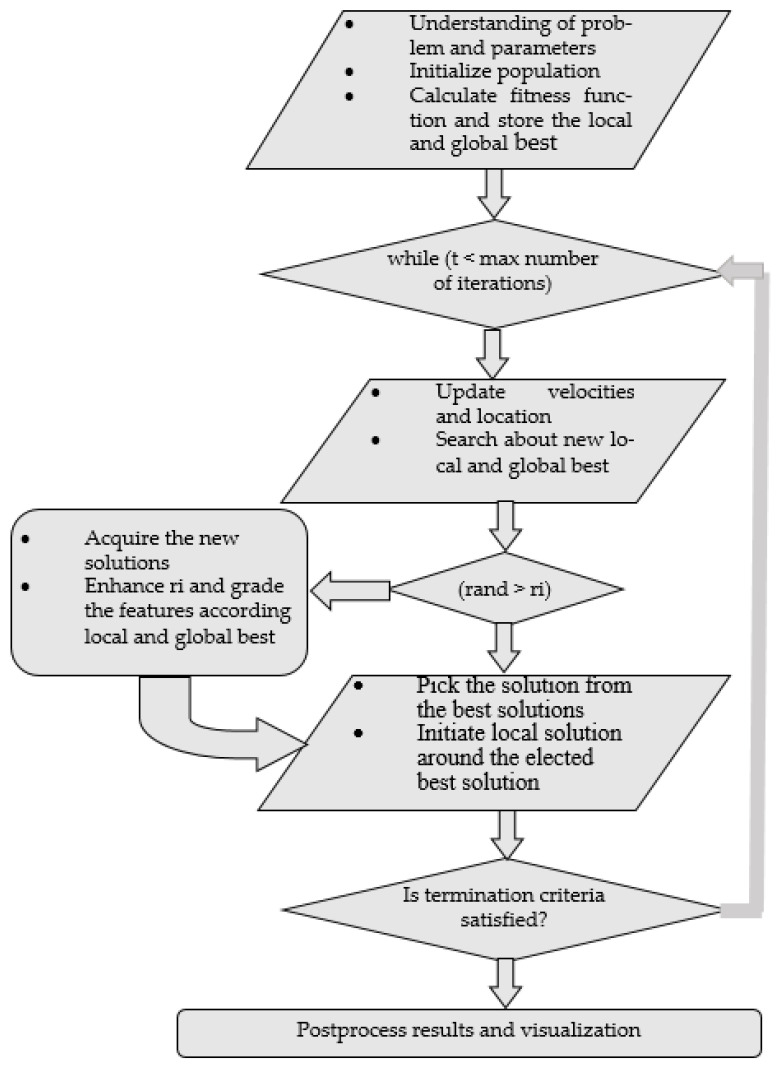
Bat optimization flow chart.

**Figure 17:**
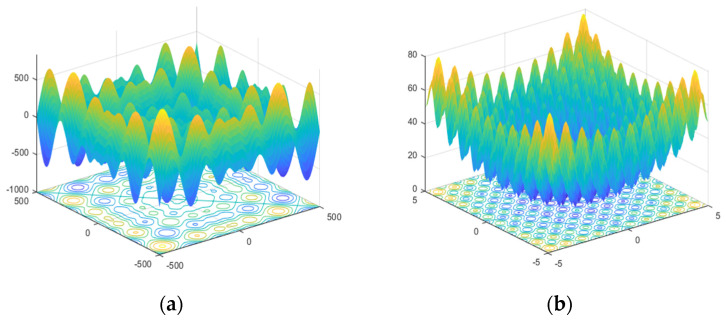
Bat optimization results. (**a**) Normal optimal features; (**b**) abnormal optimal features.

BO has multiple agents depicting the parameters of the layout dilemma, as any other metaheuristic mechanism. From real-valued vectors, the initial population is randomly generated with number *N* and dimension d by considering lower and upper boundaries using Equation (18):

(18) $$X_{ij}=X_{min}+\varphi \left(X_{max}-X_{min}\right)$$

where $X_{max}$ and $X_{min}$ are higher and lesser boundaries for dimension j,respectively, j=1, 2, . . ., d, i = 1, 2, …, and *N* and φ ranged from 0 to 1 is a randomly generated value. After population initialization, we calculated the fitness function and stored the local and the global best. We evaluated the fitness values of all humans, and, on the basis of their movements, new local and global best solutions were obtained; all the humans had velocity $Vi^{t}$ affected by a predefined frequency $f_{i}$, and finally, their new position $Xi^{t}$ was located temporally, as described in the following Equations:

(19) $$f_{i}=f_{min}+\beta \left(f_{max}-f_{min}\right)$$

(20) $$Vi^{t}=Vi^{t-1}+(Xi^{t}-X*)f_{i}$$

(21) $$Xi^{t}=Xi^{t-1}+Vi^{t}$$

where $f_{i}$ is the frequency of the *i*th human, $f_{min}$ and $f_{max}$ are lower and higher frequency values, respectively, β represents a randomly generated value, and, after comparison of all solutions, X* illustrates achieved global best location (solution). Figure 16 depicts the flow chart of the algorithm and Figure 17 represents optimization results.

#### 3.4.3. Improved Entropy Classifier

Using Shannon’s information entropy theory [53] to describe the degree of uncertainty, we proposed an improved entropy classifier for the detection of human crowd behavior. First of all, we standardized all the features using Equation (22):

(22) $$X_{ij}{}^{*}=\frac{X_{ij}-min\{X_{j}\}}{max\{X_{j}\}-min\{X_{j}\}}$$

where $X_{ij}{}^{*}$ is the value of the *j*-th feature for *i*-th human. *j* = 1,2,…,m, *i* = 1,2,…,n, while n is the count of humans and m represents the count of features. After that, the weight of *j*-th feature for *i*-th human was calculated using Equation (23):

(23) $$q_{ij}=\frac{X_{ij}{}^{*}}{\sum_{i=1}^{n}X_{ij}{}^{*}}$$

Thus, the information entropy of each feature was calculated using Equation (24):

(24) $$e_{j}=-k\sum_{i=1}^{n}\left(q_{ij}\times lnq_{ij}\right)$$

where k =$\;\frac{1}{\ln m}$. After calculating the information entropy, we then calculated the difference coefficient and maximum ratio of the difference coefficient using Equations (25) and (26):

(25) $$d_{j}=1-e_{j}$$

(26) $$D=\frac{\max\left(d_{j}\right)}{\min\left(d_{j}\right)},\;\;\;\;\;\;\;\;\;\;\;\;\;\;\;\;\left(j=1,2,\ldots ,m\right)$$

After calculating D, we then built up the scale ratio chart 1–9 using Equation (27):

(27) $$R=\sqrt[{a-1}]{\frac{D}{a}}$$

where a depicts the highest scale-value worked as an adjustment coefficient by calculating the power (a−1). The *D* is allocated to the mapping values from 1 to 9 in the above Equation. After that, from scale 1–9, mapped values were calculated, and judgment matrix R was established with elements $r_{ij}$, respectively, using Equation (28):

(28) $$r_{ij}=\frac{d_{i}}{d_{j}},\;\left(d_{i}>d_{j}\right)$$

The obtained judgment matrix satisfied the consistency test because the elements $r_{ij}$ demonstrated the ratio of difference coefficient of two features.

**Figure 18:**
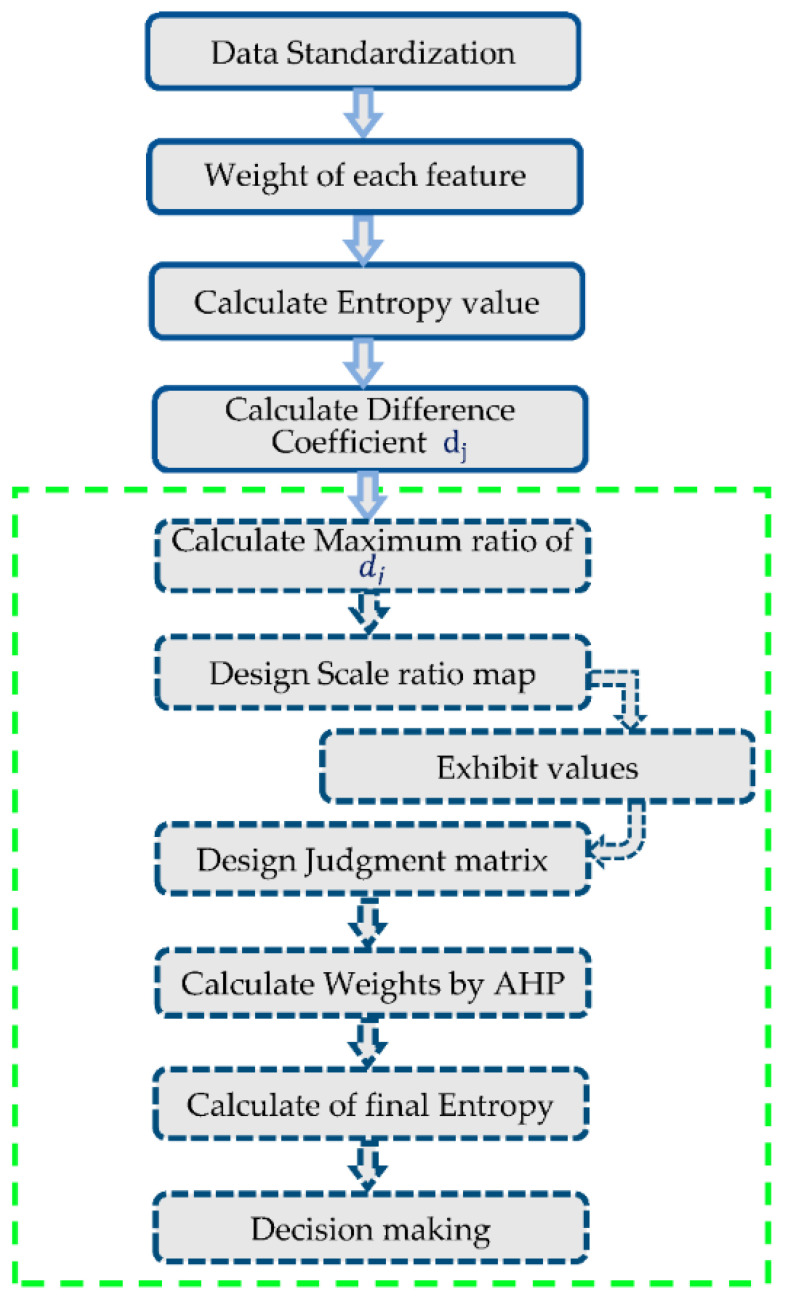
Flow chart of the improved entropy classifier.

**Figure 19:**
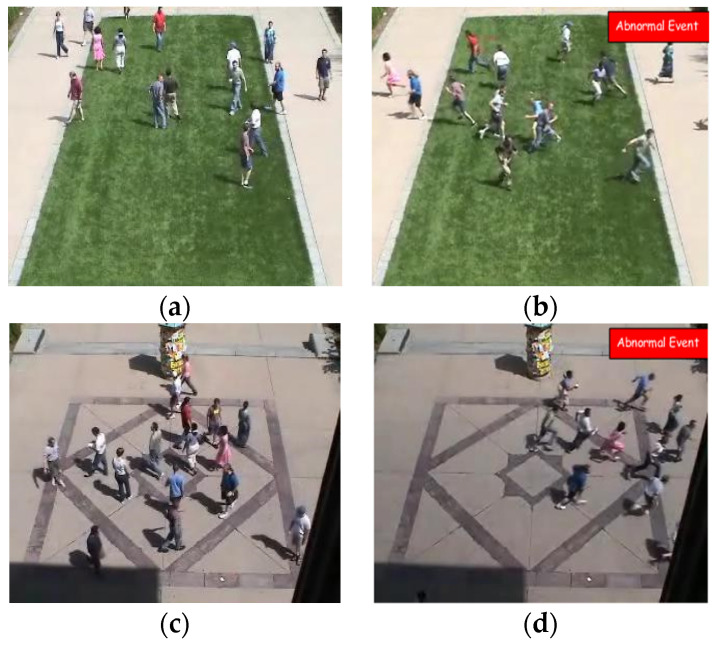
Crowd behavior detection. (**a**,**c**) Normal frames and (**b**,**d**) abnormal frames.

Thus, the consistent weights $W_{j}$ for each feature were then calculated using an analytical hierarchy process. After that, information entropy was again calculated for each feature, using these weights by utilizing Equation (24). The crowd behavior entropy of the whole system was the summary of all entropies. In this way, for every frame, the entropy value was calculated and utilized as a template. For a small entropy value less than the defined threshold, the behavior was predicted as normal; however, for entropy values higher than the set threshold, the behavior was presumed to be abnormal. A flow chart of the proposed improved entropy classifier is shown in Figure 18. Figure 19 depicts results over event classes.

## 4. Performance Evaluation

In this section, we evaluated the performance of our proposed system. We conducted experiments on two publicly available benchmark datasets to evaluate the accuracy and performance of our proposed model. The PETS2009 dataset was used to evaluate the accuracy of multi-person tracking and the UMN dataset was used to evaluate the accuracy of crowd behavior detection. We started by briefly describing the datasets used, and then the experimental results were discussed. Finally, we showed the mean accuracy of our proposed system. We also compared our proposed model with other state-of-the-art multi-person tracking and crowd behavior detection systems.

### 4.1. Datasets Description

#### 4.1.1. PETS2009 Dataset

**Figure 20:**
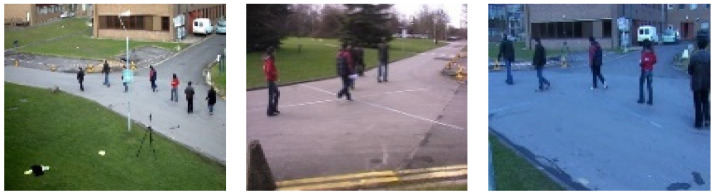
Sample frames of different synchronized views from the PETS2009 dataset.

To evaluate different video surveillance challenges, we used PETS2009, one of the publicly available benchmark datasets. The challenges included the S1 dataset for counting persons in a low-density crowd, the S2 dataset for detecting and tracking persons in medium-density crowds, and the S3 dataset for tracking and estimating the number of persons in a high-density crowd. Some sample frames of different synchronized views from PETS2009 dataset are depicted in Figure 20.

#### 4.1.2. UMN Dataset

**Figure 21:**
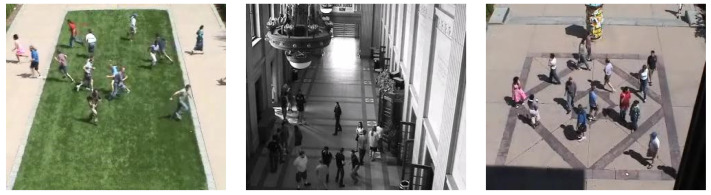
Sample frames of different scenarios of the UMN dataset.

To evaluate different video surveillance challenges for crowd behavior detection, UMN is one of the publicly available benchmark datasets. The UMN dataset consists of three different scenes, specifically, two outdoor and one indoor, with videos of 11 various panic scenarios. For the detection of abnormal behavior of a crowd, the UMN dataset is one of the best datasets that is publicly available. There were two outdoor scenes: the lawn scene, consisting of two scenarios with 1453 frames, and the Plaza scene, with three scenarios that had 2142 frames. There were six scenarios in the indoor scene, with 4144 frames. Sample frames of different scenarios of the UMN dataset are shown in Figure 21.

### 4.2. Experimental Settings and Results

We performed all the experiments on MATLAB, and the hardware system had a 64-bit intel core-i3 2.5 GHz CPU and 6 GB of RAM. Three experimental measures were used to evaluate the performance of the system: (1) mean accuracy of multi-person tracking, (2) the accuracy of human crowd behavior detection, and (3) comparisons between our proposed new system with other current and well-known systems. Experimental results showed that our proposed system produces a higher accuracy rate over existing systems.

#### 4.2.1. Experiment 1: Multi-Person Tracking over the PETS2009 Dataset

Experimental results and mean accuracy of our proposed multi-person counting and tracking model on a publicly available PETS2009 dataset are shown in Table 1 and Table 2. The ground truth was obtained by counting the number of persons in every sequence, where one sequence contained 20 frames. Table 1 depicts the mean accuracy of our proposed multi-person counting system on the first 30 sequences. As shown, the mean accuracy of our proposed model was 89.80%.

Table 2 presents the mean accuracy of our proposed multi-person tracking system. The actual number of humans is the same as for Table 1, while column 2 represents the successful tracking rate of our proposed particles force model and column 3 depicts the failure case. The mean accuracy of our proposed model for multiple person tracking was 86.95%.

#### 4.2.2. Experiment 2: Human Crowd Behavior Detection over the UMN Dataset

Experimental results using the confusion matrix and the mean accuracy of our proposed HCB model on the publicly available UMN dataset are shown in Table 3. The way to evaluate algorithms is to run them throughout a test sequence with initialization from the ground truth position in the first frame.

#### 4.2.3. Experiment 3: Multi-Person Tracking and HCB Detection Comparisons with State-of-the-Art Methods

We compared our proposed system with other well-known multi-person tracking and human crowd behavior detection methods. As depicted, our system performed better compared to existing well-known state-of-the-art methods. Table 4 shows that, in comparison to other state-of-the-art methods, our proposed system achieved an admirable accuracy rate of 86.06% for crowd behavior detection, which is higher than the accuracy of the force field model (FF) (81.04%) and the social force model (SF) (85.09%). The accuracy of other methods under the same evaluation settings was taken from [77,79].

Table 5 presents the comparison of our proposed system with other state-of-the-art systems for multi-person counting. Experiment results show that our proposed system achieved a higher accuracy rate of 89.8% over existing methods.

In Table 6, comparisons of multi-person tracking with other state-of-the-art methods show that our proposed system achieved a higher accuracy rate of 86.9% over existing methods.

## 5. Conclusions

In this paper, we proposed a new robust approach for crowd counting. We introduced and tested tracking and human behavior detection using the idea of a mutually interacting particles force model and an improved entropy classifier with spatio-temporal and particles gradient motion descriptors. Through detailed experiments, we proved the ability of the method to efficiently count, track, and detect the behavior of multiple persons efficiently in crowded scenes. The performance of our new tracking system decreases marginally with increasing numbers of persons in the scene. This is mainly due to full occlusions that occur in the test videos. We achieved promising results on the publicly available benchmark PETS2009 dataset, with an accuracy of 89.80% for multi-person counting and 86.95% for person tracking, as shown in Table 1 and Table 2. However, for HCB detection, we achieved promising results on the publicly available benchmark UMN dataset, with an accuracy of 86.06%, as shown in Table 3. Our future work will focus on some occlusion reasoning methods to further tackle the occlusion problems. We will also extend our work to multiple scene detection. We are interested in recognition of different scenes, such as sport scenes, fight scenes, robbery scenes, traffic scenes, and action scenes.

## Figures and Tables

**Table 1:** Multi-person counting accuracy over the PETS2009 dataset.

Sequence No (Frame = 20)	Actual Count	Predicted Count	Accuracy
6	3	3	100
12	4	4	100
18	5	4	80
24	6	5	83.33
30	7	6	85.71
Mean Accuracy = 89.80%			

**Table 2:** Multi-person tracking accuracy over PETS2009 dataset.

Sequence No (Frame = 20)	Successful	Failure	Accuracy
6	3	0	100
12	4	0	100
18	4	1	80
24	5	1	83.33
30	5	2	71.43
Mean Accuracy = 86.95%			

**Table 3:** Confusion matrix, showing mean accuracy for human crowd behavior detection on the UMN dataset.

Events	Normal	Abnormal
Normal	88	12
Abnormal	16	84
Mean Accuracy of Event Detection = 86.06%		

**Table 4:** Comparison of the proposed approach with other state-of-the-art methods for human crowd behavior detection on the UMN dataset.

Indoor/Outdoor Scenes	Force Field Model	Social Force Model	Proposed Method
Scene 1	88.69	84.41	87.43
Scene 2	80.00	82.35	83.21
Scene 3	77.92	90.83	90.63
Overall accuracy	81.04%	85.09%	86.06%

**Table 5:** Comparison of proposed approach with state-of-the-art multi-person counting methods.

Methods	Counting Accuracy (%)
Pixel-map based algorithm [94]	83.6
Sparsity-driven [111]	86.3
Head Shoulder based detection [100]	86.7
Skin Detection [81]	88.7
Proposed method	89.8

**Table 6:** Comparison of the proposed approach with state-of-the-art multi-person tracking methods.

Methods	Tracking Accuracy (%)
Flow Linear Programming [112]	78.8
DDPMO [113]	81.3
Appearance model [114]	83.0
Proposed method	86.9

## Data Availability

Data sharing not applicable.
